# Ancient Schwannoma of the Radial Nerve: A Case Report

**DOI:** 10.7759/cureus.42920

**Published:** 2023-08-03

**Authors:** Duy Phan, Nu Tra My Ton, Thi Hong Khang Bui, Hoang Ngoc Anh Tran, Van Tri Truong

**Affiliations:** 1 Neurosurgery, Vinmec Healthcare System, Ho Chi Minh, VNM; 2 Radiology, Vinmec Healthcare System, Ho Chi Minh, VNM; 3 Pathology, Vinmec Healthcare System, Ho Chi Minh, VNM; 4 Neurosurgery, Vinmec Healthcare System, Hochiminh, VNM

**Keywords:** radius, radial nerve, neural sheath tumor, ancient schwannoma, schwannoma

## Abstract

Ancient schwannoma is a very rare subtype of schwannoma. In this report, a case of ancient schwannoma in the upper extremity is reported. A 40-year-old man presented with a slowly growing tumor in the right forearm. He underwent surgery to remove the tumor. Investigation revealed an ancient schwannoma originated from the right radius. Careful preoperative imaging evaluation is important for correct preoperative diagnosis and surgical strategy.

## Introduction

A schwannoma is a benign tumor originating from Schwann cells. They are reported to be the most common peripheral neural sheath tumor, with 80% in some reports [1,2], and account for 8% of all soft tissue tumors [3]. Although they arise from any peripheral nerve, they are more frequently found in the upper extremity (70%) and mostly affect large nerve trunks [2,4]. Most cases can be diagnosed based on clinical presentation and imaging. Schwannoma’s histological subtypes include conventional (most common), ancient, cellular, plexiform, melanotic, intermediate, and epithelioid [5].

In this article, we report a very rare case diagnosed as an ancient schwannoma of the radial nerve. We describe the clinical presentation, imaging, histology, surgical findings, and outcome. We discuss MRI findings that support the diagnosis of ancient schwannoma.

## Case presentation

A 40-year-old man presented with an enlarging lump in the right forearm a year ago. At that time, the tumor was smaller and non-tender, and the biopsy result was a benign neural tumor. On physical examination, a 3x3 cm non-tender, firm, round, non-mobile mass in the anterior aspect of the forearm was found. The patient had no neurological deficit.

**Figure 1:**
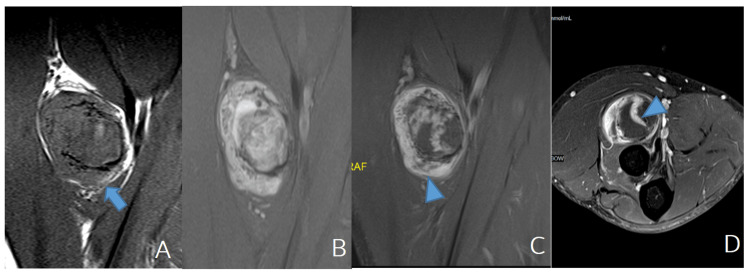
MRI imaging features: A-D: On MR, the mass is well-encapsulated with a surrounding ring of fat on T1 (arrow) (A). The lesion is primarily T1 hypointense and T2 heterogeneous (B) to hyperintense, with a fairly avid enhancement of the fibrotic capsular tissue component inside the mass on post-contrast imaging (arrowhead) (C, D). No bone marrow edema. A: MRI T1, coronal, the mass is well-encapsulated with a surrounding ring of fat on T1 (arrow); B: MRI T2, coronal; C: MRI T1 with contrast, coronal; D: MRI T1 with contrast, axial, hyperintense with fairly avid enhancement of fibrotic capsular of tissue component inside mass on post-contrast imaging (arrowhead).

His MR imaging showed a well-defined heterogeneous mass on the upper third of the right radius, 24x30x40 mm in size. On post-contrast T1-weighted imaging (T1WI), the mass showed heterogeneous enhancement with central necrosis. The tumor did not invade nearby bone or muscle (Figure 1).

**Figure 2:**
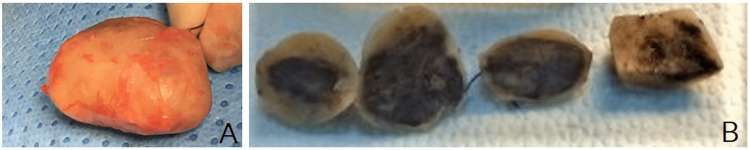
A dark yellow tumor with a fibrotic capsule was resected en bloc (A). Multiple transverse sections through the tumor that are well circumscribed and display foci of hemorrhage and cyst formation (B) A: a dark yellow tumor with a fibrotic capsule was resected en bloc; B: multiple transverse sections through the tumor that are well circumscribed and display foci of hemorrhage and cyst formation.

These findings suggested a right radial nerve schwannoma. Since the tumor increased in size significantly, removal surgery was suggested. The mass was in the anterior aspect of the radial bone, 3x3x4 cm in diameter, dark yellow in color (Figure 2A).

**Figure 3:**
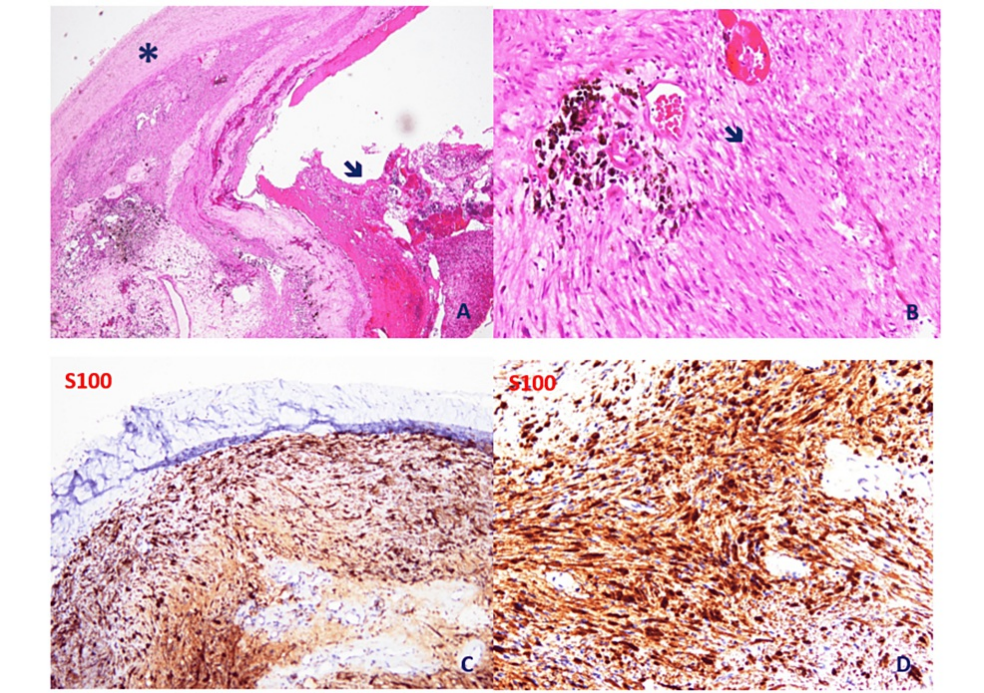
An ancient schwannoma with a thick capsule (*), cystic degeneration and hemorrhage (ancient change) (arrow) (A); hemosiderin deposition (*), nuclear palisading (Verocay bodies) (arrow) (B); immunohistochemistry with S100 strong and diffuse staining (C), nuclear atypia with S100 positive (*) (D). A: ancient schwannoma with a thick capsule (*), cystic degeneration, and hemorrhage (ancient change) (arrow); B: hemosiderin deposition (*), nuclear palisading (Verocay bodies) (arrow); C: immunohistochemistry with S100 strong and diffuse staining; D: nuclear atypia with S100 positivity (*).

A linear incision in front of the forearm was used. In the operation, the tumor was easily detached because there was no definite adhesion to adjacent tissue. The tumor, which originates from the superficial branch of the radial nerve, was then removed en bloc. Inside the mass, some small hemorrhagic foci were found (Figure 2B). The patient recovered uneventfully from the surgery and was discharged from the hospital the same day. The pathological features showed an encapsulated tumor with nuclear atypia (without mitosis), cystic degeneration, hemorrhage (ancient change), and a strong positive for S-100, which was consistent with a diagnosis of ancient schwannoma (Figure 3).

## Discussion

The presented tumor contained fluid and hemorrhage, in terms of imaging. This can be misdiagnosed with other malignancies such as malignant fibrous histiocytoma, malignant peripheral nerve sheath tumor, fatty sarcoma, synovial sarcoma, etc. Although only a few cases of ancient schwannoma do occur at the elbow have been found in the literature, physicians need to take into account the possibility of ancient schwannoma in the present patient [6] because the surgical strategy may be different between a malignant tumor and a benign one.

On magnetic resonance imaging, an ancient schwannoma tumor will be a mass with a heterogeneous signal, depending on the ratio between the Antoni A and Antoni B regions. The lesion is hypointense on T1W, hyperintense on T2W corresponding to the Antoni B region, T2 low- to intermediate signal, and avid enhancement corresponding to the Antoni A region [7,8].

The fibrotic capsule is the hypointensity margin on T1W and T2W, which enhances post-contrast administration. According to Isobe et al., the fibrotic capsule and internal heterogeneity are important to distinguish schwannoma from other tumors [9]. Our case has both of the above findings.

The split fat sign is commonly present in deep schwannomas because each neurovascular bundle is usually surrounded by surrounding fat tissue, so when the tumor grows slowly, there is often a fat border. While malignant peripheral nerve sheath tumors will often invade or infiltrate adjacent fatty tissue, this sign is not present. This is not a specific yet common sign [6-8]. Our case also possesses this feature.

In summary, one case of ancient schwannoma of the radial nerve has been described. To the best of our knowledge, there are only two case reports in the literature [10]. Two critical features that help differentiate ancient schwannoma from malignant tumors are the fibrotic capsule and split-fat sign.

## Conclusions

Ancient schwannoma is rare. We report the third case of an ancient schwannoma in the upper extremity. A careful preoperative imaging evaluation is very important so that we may have a correct diagnosis and surgical strategy.
